# Plant-Derived Anticancer Agents: Lessons from the Pharmacology of Geniposide and Its Aglycone, Genipin

**DOI:** 10.3390/biomedicines6020039

**Published:** 2018-03-26

**Authors:** Solomon Habtemariam, Giovanni Lentini

**Affiliations:** 1 Pharmacognosy Research Laboratories, University of Greenwich, Central Avenue, Chatham-Maritime, Kent ME4 4TB, UK; 2 Herbal Analysis Services, Chatham-Maritime, Kent ME4 4TB, UK; 3 Department of Pharmacy-Drug Sciences, University of Studies of Bari Aldo Moro, Via E. Orabona n. 4, 70126 Bari, Italy; giovanni.lentini@uniba.it

**Keywords:** cancer, carcinogenesis, metastasis, genipin, geniposide, reactive oxygen species, apoptosis, uncoupling protein 2

## Abstract

For centuries, plants have been exploited by mankind as sources of numerous cancer chemotherapeutic agents. Good examples of anticancer compounds of clinical significance today include the taxanes (e.g., taxol), vincristine, vinblastine, and the podophyllotoxin analogues that all trace their origin to higher plants. While all these drugs, along with the various other available therapeutic options, brought some relief in cancer management, a real breakthrough or cure has not yet been achieved. This critical review is a reflection on the lessons learnt from decades of research on the iridoid glycoside geniposide and its aglycone, genipin, which are currently used as gold standard reference compounds in cancer studies. Their effects on tumour development (carcinogenesis), cancer cell survival, and death, with particular emphasis on their mechanisms of actions, are discussed. Particular attention is also given to mechanisms related to the dual pro-oxidant and antioxidant effects of these compounds, the mitochondrial mechanism of cancer cell killing through reactive oxygen species (ROS), including that generated through the uncoupling protein-2 (UCP-2), the inflammatory mechanism, and cell cycle regulation. The implications of various studies for the evaluation of glycosidic and aglycone forms of natural products in vitro and in vivo through pharmacokinetic scrutiny are also addressed.

## 1. Introduction

For generations, plants have been extensively used by mankind to treat a number of diseases, including cancer. Many exemplary anticancer chemotherapeutic agents of plants origin with distinct biochemical mechanisms are also in use today. The most popular plant-derived anticancer compounds of clinical significance include those specifically targeting the cellular microtubule cytoskeleton system such as the taxanes (e.g., paclitaxel (Taxol) and docetaxel (Taxotere)), vincristine and vinblastine [1]. The dynamic process of microtubule assembly and disassembly involved in diverse cellular processes such as intracellular transport (e.g., exocytosis), cell division, and cell motility (or migration) could be hindered by such drugs, leading to cell growth arrest and induction of cell death. Numerous other drugs of plants origin act through the same mechanism, among which colchicine, combretastatin, and taccalonolides have been utilized in various forms. Podophyllotoxins and other cancer chemotherapeutics derived from such structural skeleton (e.g., epipodophyllotoxins including etoposide and teniposide) that act by inhibiting topoisomerase II are further remarkable examples of anticancer drug discoveries based on natural products/plants. Another group of plant-derived therapeutic agents are the cytotoxic quinoline alkaloids, such as camptothecin, which inhibit topoisomerase I. Review articles on the mechanism of action of such plant-derived chemotherapeutic agents are widely available [2,3].

With increased knowledge and understanding of cancer biology, to date, there are numerous options for cancer therapy. In most cases, solid tumours or solid masses are removed when possible by surgical means followed by radiotherapy. Chemotherapy, however, remains the most common therapeutic option, and developments in the last few decades have even added several biological agents (e.g., trastuzumab (Herceptin) and bevacizumab (Avastin)) [4], although the cost of such treatments is often beyond the reach of many patients. Hormone and immunotherapy options are also available. Further development of new drugs for cancer therapy in recent years has not stalled but rather accelerated. In 2014, it was reported that 771 new agents were in the pipeline, while the number of new drugs that had received regulatory approval by the Food and Drug Administration (FDA) since 2011 were 55 [5]. According to some reports (e.g., [6]), there were at least 16 drugs for oncology approved by the FDA in 2017. A similar high number of approvals has also been reported in Europe, but the rather fast rate of drug approval in the recent years has not brought a real breakthrough in cancer chemotherapy. For example, the European Medicines Agency (EMA) is reported to have approved the use of 48 cancer drugs for 68 indications from 2009 to 2013. According to a recent report by Davis et al. [7], however, most of these drugs did enter into the market without evidence of benefit for patients’ survival or quality of life. In a study following 3.3 years after their market entry, the potential of these drugs to extend or improve life for most of the indicated cancer cases was not validated [7]. The toxic side effects of chemotherapeutic agents along with the development of resistance coupled with metastasis, non-selectivity, bioavailability, and rapid clearance, are among the common drawbacks of the existing available drugs.

For the above-mentioned reasons, there is still enormous appetite to discover new drugs from natural and synthetic sources. The grim reminder is also that cancer remains the leading cause of morbidity and mortality in the world with approximately 14 million new cases reported in 2012 [8]. Being the second leading cause of death globally, responsible for 8.8 million deaths, cancer was also the second most leading cause of death in the world in 2015. The vast majority of mortality (~70%) from cancer occurs in less developed countries where the cost of therapy is beyond the reach of many patients [8]. Whether one aims to discover novel drugs or validate those natural sources (e.g., plants) commonly used as traditional medicine in developing nations, scrutinizing decades of research on those reported to show some promise is a vital scientific endeavor. In this regard, the present review highlights the lessons learned from researches on the iridoid glycoside, geniposide, and its aglycone, genipin.

## 2. Natural Sources of Geniposide and Genipin

**Figure 1:**
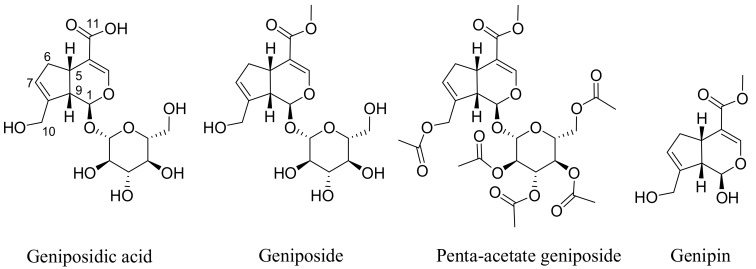
Structures of geniposide and its analogues. Geniposide is a natural analogue or methyl ester of geniposidic acid. Genipin is the aglycone of geniposide which is also present in plants, while penta-acetyl geniposide is a synthetic derivative widely employed in anticancer activity studies.

The most widely reported sources of geniposide (Figure 1) are the fruits of *Gardenia jasminoides* Ellis (Rubiaceae) that has been used in traditional Chinese medicine for centuries. Numerous other species of the genus and other members of the family Rubiaceae have been known to contain geniposide. A review article on the natural occurrence of geniposide comprising around 34 different species has appeared recently [9]. The hydrolysis product of geniposide, genipin (Figure 1), is also found along with geniposide and several derivatives (e.g., geniposidic acid). Other structural analogues of the iridoid skeleton as well as compounds derived from glycosylation and further esterification with aromatic acids have also been isolated from various plants in the last few decades [9].

The identification of geniposide as a new iridoid glycoside from *G. jasminoides* goes as far back as the 1960s [10,11]. Numerous pharmacological activities of genipin and geniposide have been reported since then, and some review articles on their antidiabetic and neuroprotective effects (e.g., in Alzheimer’s diseases) have been published by our laboratories [12,13]. The present critical review highlights progress in research with respect to the anticancer potential of geniposide and its aglycone genipin. The two common acclaimed medicinal plant sources of these compounds in the various literature articles reviewed herein are the fruits of *G. jasminoides* and *Gardenia *Fructus** (San-jee-chee in Chinese), which are highly cited for their medicinal uses. In these plants, geniposide is also a major component and serves as a quality control marker of crude plant drug preparations. For example, Yin et al. [14] have shown that geniposide accounts for 72.58–88.27% of the total components of extracts obtained from the dried and ripe fruit of *G. fructus* collected from various regions of China.

## 3. Physicochemical Properties and Associated Pharmacokinetics Profile

With a molecular formula of C_17_H_24_O_10_, geniposide (Figure 1) is a small-molecular-weight (388.366 Da) compound. The presence of a sugar (glucose) moiety in the molecule gives the compound its polarity with good water solubility and hence better expected bioavailability compared to its aglycone (genipin). The partition coefficient (P) of geniposide on the basis of the octanol/water system is reported as 0.1077, while its log *p* value is −0.97 [15]. This suggests that the expected rate of absorption in the small intestine would be poor, as the compound may not be readily passing through cell membranes. The absorption of geniposide from the crude extract of *G. fructus* in the rat intestine was studied by Zhang et al. [15], and the reported absorptive rate constants (K) at the concentration of 0.078, 0.311, 0.780 g/L were 0.130, 0.056, and 0.031 h, respectively. This absorption was considered poor although the compound was taken up in all small intestinal segments of rats, and the highest levels of absorption were in the duodenum.

Yang et al. [16] studied the pharmacokinetics profile of geniposide after administration through four routes in rats. The absolute bioavailability was reported as follow: F (i.g.) = 9.74%, F (intranasal, i.n.) = 49.54%, and F (intramuscular) = 72.69%, respectively. The pharmacokinetic profiles of geniposide following oral administrations of the pure compound and in crude herbal products were also studied in rats and in vitro using Caco-2 cells [17]. It was reported that geniposide had a better absorption in the duodenum and jejunum in vivo through passive diffusion. While geniposide might be the potential substrate for P-glycoprotein as assessed by both models, an enhancement of absorption was noted when the drug was administered in the crude (herbal) rather than in the purified form. After the oral administration of a *G. fructus* extract containing 50 mg/kg of geniposide, the mean C_max_ of geniposide was 0.68 μg/mL at 0.44 h, the mean area under the plasma drug concentration-time curve (AUC) was 1.46 μg/mL/h (0.024 μg/mL/min), while the mean apparent t_1/2_ was estimated to be 0.94 h [18].

A study by Li et al. [19] tried to address the transformation of geniposide after absorption in rats. The authors reported the detection of 17 metabolites in the plasma, 31 in the urine, 6 in the heart, 12 in the liver, 3 in the spleen, 6 in the lung, 12 in the kidney, 6 in the brain, and 4 in the liver microsomes. The transformation of geniposide included hydrolysis, hydroxylation, taurine conjugation, hydrogenation, decarboxylation, demethylation, sulfate conjugation, cysteine *S*-conjugation, and glucosylation. Lu et al. [20,21,22] also conducted comparative bioavailability studies on geniposide in mice after i.n., intragastric (i.g.), and intravenous (i.v.) administration. They reported bioavailability as 85.38% and 28.76% for i.n. and i.g., respectively, when borneol was used as a vehicle. The i.v. administration, which does not involve any absorption mechanisms, was the best in making the drug available to tissues. The reported AUC_plasma_ of i.v., i.n., and i.g. were 324.88, 277.39, and 93.44 μg/mL/min, respectively [22].

**Figure 2:**
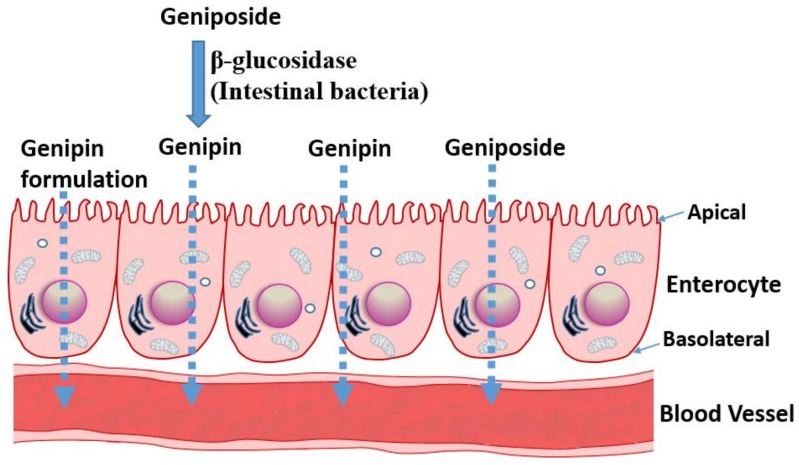
Transport mechanisms of geniposide and genipin. The absorption of geniposide, which is predominantly present in plant extracts, could be enhanced by transformation in the gut through the action of bacterial β-glucosidase enzymes. Other preparations, such as borneaol or crude plant extracts, could increase the absorption from the gut. The bioactive molecule, genipin, is highly non-polar and water insoluble, and a formulation strategy is required to maximize its absorption.

All the above data suggest that geniposide has somehow a poor absorption profile but could be readily taken up and distributed in animal tissues. The various pharmacological effects demonstrated in animal models (reviewed in [12,13]) and discussed in the following sections also suggest that the compound and/or its metabolites have profound effects after administration through various routes. The pharmacokinetic profile of its aglycone, genipin, is however not readily available for comparison, although few pioneering studies of significance are worth mentioning. A study by Ako and Kobashi [23,24] have shown that geniposide, once orally administered, is converted to genipin in the intestine, which acts as the active principle. β-d-glucosidases activities of the intestinal bacteria were implicated in this transformation. A study by Yim et al. [25] further established that the transformation of glycosides into a bioactive aglycone form is extended to other natural products, such as ginsenoside Rb1, glycyrrhizin, and baicalin. In our recent review article on rutin and its aglycone, quercetin, as potential therapy for inflammatory bowel diseases, the transformation of a glycoside form to a bioactive aglycone molecule in the intestine was highlighted [26]. When quercitrin was anaerobically incubated with human intestinal bacteria, the main product was also found to be quercetin [27]. Moreover, a range of reactions, including hydroxylation, demethylation, deglycosylation, and ring-cleavage can occur under the action of intestinal bacteria, and compounds like quecetrin can give rise to bioactive molecules such as 3,4-dihydroxybenzoic acid [28]. Hence, intestinal transformation and absorption (Figure 2) must be considered from the outset when one is assessing the anticancer potential of geniposide and its aglycone. In this regard, a study by Kang et al. [29] is a further example elaborating this therapeutic principle. They have reported that the aglycone genipin is much more cytotoxic to human hepatoma HepG2 cells than geniposide. Moreover, the metabolic activation system for geniposide was confirmed to be the passage through the intestine, as human intestinal bacterial cultures (*Bifidobacterium longum* HY8001 or *Bacteroides fragilis*) or fecal preparations could activate geniposide to kill cancer cells. The absorption of geniposide into the blood stream in its intact form could also be augmented if antibiotics were used to suppress the activity of intestinal bacteria [30].

The hydrolysis of geniposide to genipin by β-glucosidase could also be studied directly by using purified enzymes. Hence, the immobilized glycosyl hydrolase family 3 β-glucosidase has been effectively used to convert geniposide in a hot-water extract of *G. fructus* into genipin [31]. With respect to probiotic applications, the role of lactic acid bacteria (*Lactobacillus* sp.) in the intestine in food transformation and hydrolysis of glycosides (e.g., ginsenoside Rd and glucosidic isoflavones) using β-glucosidase are well understood [32,33]. Furthermore, *Lactobacillus rhamnosus* GG strain (LGG) has been shown to enhance the in vitro anticancer effects of geniposide [34].

The above data have profound implications on the extraction of bioactive compounds from natural sources, drug formulation, and pharmacokinetic and/or pharmacodynamics parameters. For example, a drug molecule of nonpolar nature that is of interest in vitro could be rather administered in its glycosidic form not only to increase its bioavailability but also to convert it to its more bioactive form in the intestine. The extraction of compounds by non-polar solvents that may give a good bioactivity in vitro may also lead to non-bioactive molecules in vivo, given the biotransformation issue discussed above. As shown in the following sections, the relative potency of genipin and geniposide varies: while genipin is profoundly more active in vitro, its concentration in many plants (for example in *G. fructus*) is far lower than that of geniposide. Hence, the anticancer effect of such plant extracts is partly a result of the biotransformation of the orally administered preparations in the gut, and, hence, future anticancer drug development prospects should consider enzyme-catalyzed tools in converting glycosides into bioactive aglycones (Figure 2).

## 4. Anticancer Effects of Geniposide and Genipin

### 4.1. Direct Cytotoxic Effect on Cancer Cells

The assessment of direct cytotoxicity on cancer cells, often in combination with the measurement of apoptosis events, are common in vitro assays employed to show potential anticancer effects of natural products. Genipin and geniposide have been shown to be cytotoxic in numerous cancer cell types including colorectal cancer [35,36], pancreatic adenocarcinoma cells [37], AGS and SNU638 human gastric carcinoma cells [38,39,40], non-small-cell lung cancer H1299 cells [41], prostate (DU145 and PC3) cancer cells [42,43,44], hepatocarcinoma (HepG2 and Hep3B) cells [45,46] breast cancer (MDA-MB-231) cells [47], human leukaemia (K562, HL-60, U266, U937) cells [48,49,50], and tongue squamous carcinoma (HSC-3) cells. The acetylated product of geniposide, penta-acetyl geniposide (Figure 1), has also been investigated and has shown in vitro cytotoxicity in a range of cell lines, such as C6 glioma cells [51,52,53,54,55,56,57].

While the in vivo effect of compounds following oral administration could be variable, depending on intestinal transformation, direct cytotoxicity or induction of apoptosis are often more pronounced for aglycones than for their glycosidic analogs. For example, the flavonoids quercetin and myricitin are more potent in the induction of apoptosis in cancer cells than their corresponding glycosides quercitrin and myricitrin, respectively [58,59]. Despite some unique and general mechanisms (see following sections) attributed to the anticancer effects of geniposide and genipin in various cancer cells lines, we should be cautious about their value as anticancer lead compounds. The effective dose of geniposide is far higher than 100 μM and hence should be considered weak. Even for its acetate derivative, which is considered more active, many studies used 200 or 300 μM as effective doses (e.g., [55,60], while some studies even used 600 μM (e.g., [53]). On the other hand, for the anticancer compound genipin (more active in vitro than geniposide), which displays cancer growth inhibition at a lower micromolar range concentrations, the most effective doses shown in various studies remain around 100 and 200 μM [35,37,42,45,48,61]. In this regard, the IC_50_ of genipin in H1299 cells was 351.5 μM [41]. In the search of novel potential anticancer agents from natural and other sources, activities (IC_50_ values) in nanomolar ranges are often considered potent, while those in the submicromolar or micromolar ranges are considered promising. The various plant-derived cytotoxic agents of clinical significance fall within this last category. For example, the IC_50_ value of paclitaxel in various human tumour cell lines range between 2.5 and 7.5 nM) [62]. In our laboratories, many terpenoids that merited a report as potential anticancer agent have an IC_50_ lower than 20 μM and are considered very active when an activity far lower than 10 μM is achieved [63,64,65,66]. An activity up to 50–100 μM may be considered moderate in view of potent compounds, such as the podophyllotoxin analogues, that we have isolated from natural sources with a potency in the nanomolar range and that comparable to taxane [67]. Considering these scenarios, the reported anticancer effect of geniposide is nothing but weak. The mismatch between the high number of publications on natural products with potential anticancer effect and the number of those products making it into the development stage is thus partly due to the poor intrinsic potency these compounds are endowed with. For example, many terpenoid compounds that we showed to have general cytotoxicity in cancer cells possess one or few α,β-unsaturated carbonyl groups that could initiate a Michael-type addition reaction leading to cell damage and/or cytotoxicity. To date, many α,β-unsaturated carbonyl compounds are known to display tumour-specific cytotoxicity through Michael acceptor mechanisms, and their activity could also be reversed by *N*-acetylcysteine [68]. Geniposide and genipin possess this structural moiety (Figure 1), and, even though multiple mechanisms (see next section) are implicated, such general mechanism could be implicated in their rather weak action as cytotoxic agents in cancer cells. In fact, their cytotoxicity and other biological effects that are mediated via the generation of reactive oxygen species (ROS) have been shown to be reversed by an antioxidant such as *N*-acetylcysteine [69]. Moreover, compounds containing such structural moiety are also known to display antibacterial effects against the common gram-positive bacteria [70,71,72], an effect that is shared by genipin/geniposide [73]. Keeping in mind that the general mechanism of the above-mentioned structural moiety is evident in biological systems [74], the in vivo anticancer effect of these compounds is also demonstrated. For example, when C6 glioma cells were inoculated into rats, some tumour growth inhibition was observed after treatment with penta-acetyl geniposide at doses of 5 and 10 mg/kg [56]. The most pronounced effect was, however, observed when the drug was administered as a pre-treatment, for which the latency period T_50_ (time for 50% tumour incidence) was prolonged. The reported growth inhibition at week 7 of treatment with the two doses was 41% and 75%, respectively [56].

### 4.2. Effects on Carcinogenesis

As discussed above, doses as small as 5 and 10 mg/kg of penta-acetyl geniposide have been demonstrated to increase the latency of tumour development in animals. Lee et al. [75] investigated the potential effect of the topical application of geniposide on 12-*O*-tetradecanoylphorbol-13-acetate (TPA)-induced promotion of skin tumours in mice previously initiated with benzo[a]pyrene. In this model, geniposide (0.2 or 1.0 μmol) administered with TPA (15 nmol) twice weekly for 20 weeks was reported to suppress tumour growth by 84% or 89%, respectively. In the same model, geniposide also inhibited the induction of epidermal ornithine decarboxylase activity by TPA (5 nM), as well as skin inflammation (TPA-induced oedema of mouse ears by 41% or 43%, respectively). Other markers of inflammation induced by TPA in the mouth skin, such as hydrogen peroxide (H_2_O_2_) and myeloperoxidase, were also suppressed. In another study by Wang et al. [76], the inhibitory effect of geniposide on aflatoxin B_1_ (AFB_1_)-induced DNA repair synthesis in primary cultured rat hepatocytes were investigated. The authors showed that geniposide could suppress the AFB_1_-induced DNA repair synthesis through an increased AFB_1_ detoxification metabolism. Hence, the activities of glutathione (GSH)-*S*-transferase (GST) and GSH-peroxidase (GPx) in AFB_1_-treated cultured cells were shown to be enhanced by geniposide. Other studies on cultured AFB_1_-treated C3H10T12 cells showed that penta-acetylated geniposide could interfere with the aflatoxin-induced DNA damage and repair processes [77]. In line with these in vitro effects, Wang et al. [78] also demonstrated that geniposide could suppress hepatic AFB_1_–DNA binding and AFB_1_ hepatotoxicity in rats. Serum marker enzymes of the liver, such as aspartate aminotransferase (AST), alanine aminotransferase (ALT), and γ-glutamyltranspeptidase (γ-GT) were elevated following treatment with geniposide (10 mg/kg) daily for three consecutive days. These data, along with the amelioration of the AFB_1_–DNA adduct formation by geniposide [78], is in line with the suggested chemopreventive properties and/or anticarcinogenic potential of geniposide. Lin et al. [79] also assessed the potential of this compound in suppressing the development of γ-glutamyl transpeptidase (GGT)-positive foci induced by AFB_1_ in rats. The rational of the experimental model appears to be related to GGT inhibition being a target of AFB_1_-induced hepatocarcinogenesis. Since pioneering studies proposing a crucial role for GGT in cancer development in the 1980s, it potential as a target in various stages of cancer progression has been established. Hence, some competitive and non-competitive inhibitors, including the glutamate analogs, have been shown to be promising as potential anticancer agents [80,81,82]. Upon exposure to AFB1 (like in cases of diet contaminated with aflatoxins), an increase in the GGT-positive foci have been widely reported, while the activity of the enzyme in hepatocytes in normal individuals is maintained at a very low level. Data by Lin et al. [79] revealed a suppressive effect of geniposide on the AFB1-induced GGT-positive foci (with a diameter larger than 0.3 mm). More importantly, the doses employed (1 and 2 mg/kg, p.o.) to achieve this outcome were very low. This effect is related to the pro-oxidant/antioxidant effect of geniposide discussed in the following sections. Located on the cell surface, GGT hydrolyzes the extracellular GSH and increases the intracellular amino acid pool for GSH synthesis. The high level of ROS in cancer cells could thus be mitigated through the action of GGT, a mechanism which is crucial for carcinogenesis and drug resistance to chemotherapeutic agents. Excellent review articles on GGT physiology and pharmacology are available [83,84]. As with geniposide, many natural products, such as green tea epicatechins, have been shown to act through GGT inhibition to induce their antimutagenic and anticarcinogenic effects [85]. The anticarcinogenic mechanism of genipin/geniposide could also be attributed to the general antioxidant mechanism that is common to many natural products. The induction of nuclear factor-erythroid-2-related factor 2 (Nrf2) antioxidant enzyme along with GPx by genipin has been shown in AGS cell line [39]. This antioxidant effect was, however, shown at smaller doses (less than 25 μM) of genipin treatment, which were also associated with C-Jun-NH2-kinase (JNK) activation by the compound. Hence, readers should note that such an action may not be evident at the high doses of genipin mediating anticancer effects.

Another mechanism by which these compounds may exert anticancer activity could be associated with their antiviral effects. A study by Son et al. [86] demonstrated that genipin (70 μM) can have antiviral effects against Epstein–Barr virus (EBV). As EBV causes several human cancers [87,88], an antiviral effect could have implications in the protection against virus-induced carcinogenesis. A study by Cho et al. [89] also showed an effect of genipin against the Kaposi’s sarcoma-associated herpesvirus (KSHV). The IC_50_ for genipin was, however, reported as 49.5 μM in iSLK-puro (KSHV-negative) cells and 72.5 μM in iSLK-BAC16 (KSHV-positive) cells. Hence, its effect in this viral system is not in favour of antiviral effect, but to the contrary, it could promote KSHV latent replication at lower concentrations [89]. By using the murine model of influenza respiratory tract infection, Zhang et al. [90] demonstrated the antiviral effect of geniposide against pandemic A/Jiangsu/1/2009 (H1N1) influenza virus. The antiviral effect of geniposide against EV71 virus has also been reported [91]. Hence, even though further research is required, some of the reported antiviral effects could be implicated in the anticarcinogenic activity of genipin and geniposide analogs.

### 4.3. Effects on Cancer Metastasis

Wang et al. [46] studied the anti-metastatic potential of genipin in human hepatocellular carcinoma cells in vitro. They showed, through an orthotopical implantation model, that genipin could suppress the formation of intrahepatic metastases as well as tumour expansion in the liver at its non-toxic concentrations (60–120 μg/mL). Cell motility and invasiveness through extracellular matrix (ECM) were also inhibited by genipin. The authors also presented an interesting insight into the mechanism of action of genipin. While the expression levels of matrix metalloproteinase-2 (MMP-2) (mRNA or protein) were not affected, genipin was shown to upregulate the expression of the endogenous inhibitor of MMP-2, i.e., tissue inhibitor of matrix metalloproteinase-1 (TIMP-1). This effect of genipin was also correlated with the activation of p38 mitogen-activated protein kinase (MAPK) signaling, which appeared to be correlated to apoptosis induction by genepin in cancer cells (see below for mechanisms of action). A study by Huang et al. [92] on the penta-acetylated geniposide also showed anti-metastatic potential of this compound in rat neuroblastoma (C6 glioma) cells, wherein inhibitory effects were observed in cell-matrix adhesion, wound healing, and Boyden chamber assays. In agreement with the report by Wang et al. [46], these researchers also observed a decreased activity of MMP-2 in a gelatin zymography assay and increased levels (mRNA) of TIMP-2; however, in contradiction with this, reduced mRNA levels of MMP-2 and of membrane type I matrix metalloproteinase (MT1-MMP) were reported [92]. Another inhibitory effect of penta-acetylated geniposide was observed on the protein expression of phosphoinositide 3-kinase (PI3K), the phosphorylation of extracellular signal-regulated kinases 1 and 2 (ERK1/2), and the activation of transcription factor nuclear factor κB (NF-κB), c-Fos, and c-Jun. Readers should note that the in vitro cell migration assays were conducted at concentrations of 0.15 or 0.3 mM, and the best effective dose of 0.3 mM employed (non-toxic concentration) should be considered rather high. Other studies have shown that genipin could suppress the invasive/migratory abilities of the highly invasive MDA-MB-231 human breast cancer cells, suggesting a potential effect in breast cancer metastasis [47].

The inhibitory effect of genipin on vascular smooth muscle cell proliferation and migration through tumour necrosis factor-α (TNF-α) suppression has been studied by Jiang et al. (2013) [93]. They have shown that this effect was mediated through the induction of haem oxygenase-1 (HO-1), the expression/activation of ERK/MAPK, and the phosphorylation of protein kinase B (PKB or Akt), without a significant effect on p38 MAPK and JNK. In their assay, the generation of ROS by TNF-α was also blocked. The study by Kitano et al. [94] on the anti-fibrogenic effect via decreasing TGF-β1 expression in human sub-conjunctional fibroblasts has also implications on wound healing and cancer metastasis. They showed that genipin could suppress wound-induced cell migration and proliferation of fibroblasts by decreasing collagen type I (mRNA and protein), transforming growth factor β1 (TGFβ1), and α-smooth muscle actin (αSMA) expression. Smad2 (mothers against decapentaplegic homolog 2; also known as SMAD family member 2) signaling was also inhibited by genipin [94].

On the basis of the above reported findings, multiple mechanisms appear to be involved in the potential cancer growth and metastasis inhibitory effects of genipin. First, the solid tumour mass must disintegrate to release cancer cells that would travel and invade new tissues to establish foci of cancer cells. This requires the degradation of adhesion molecules (e.g., cadherins) that keep cells together in tissues (reviewed in [95]). ECM degradation by a range of proteolytic enzymes, including matrix metalloproteinases (MMPs), is another important feature of cancer metastasis [96,97,98]. During metastasis and its related pathological process, angiogenesis, ECM degradation results in the activation and/or release of various mediators that increase cell proliferation, invasion, and angiogenesis. The migration of cancer cells through ECM, blood media, and tissues mirrors that of leucocyte infiltration into extravascular tissues under inflammatory conditions. Hence, several key molecular and biochemical targets are shared by these two processes. The modulation of cell-cell and cell-matrix interactions is thus fundamental for a cancer cell to migrate, invade, or escape from destruction by the immune system. The MMPs, through their zinc endopeptidases action, degrade a plethora of proteins ranging from proteinases, MMPs themselves, proteinase inhibitors, growth factors, chemokines, cytokines, and various cell adhesion molecules. Hence, the potential of genipin to modulate cancer metastasis through effects on MMPs appears to be established. Another feature of metastasis overlapping with inflammation is the signal transduction pathway mediated by NF-κB. Many natural products have been shown to modulate cancer metastasis through an effect on the NF-κB mobilization [99]. As demonstrated for genipin above, a number of studies have also shown that the activation of MMPs (MMP-1, -2, -3, and -9) is regulated by the PI3K, NF-κB, and AP-1. As with inflammation and angiogenesis, cancer metastasis has also been shown to involve MAPK (e.g., JNK, p38, and ERK), which appear to be regulated by genipin. More data on angiogenesis including the effect on key mediators, such as the vascular endothelial growth factor (VEGF), and in vivo evidence are needed to further validate the potential of these compounds as anti-metastatic agents.

**Figure 3:**
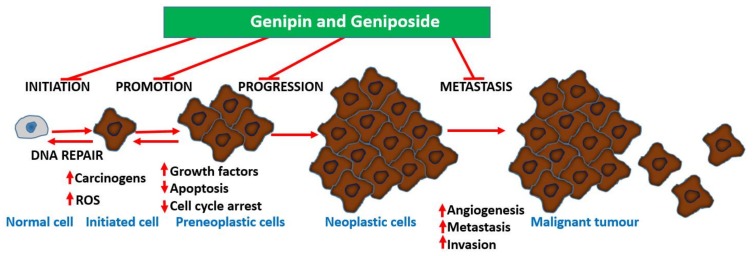
The various stages of cancer development and their potential modulation by genipin and analogs. Genipin/geniposide appear to target almost all stages of cancer development via multiple mechanisms.

Undoubtedly, multiple mechanisms of action take part in the anti-metastatic effect of geniposide/genipin and of the crude extract preparations of plants that produce them. Following the demonstration of the potent anti-angiogenic activity of *G. jasminoides* Ellis in the chick embryo chorioallantoic membrane assay, a bioassay-guided isolation study identified geniposide as the active principle [99]. In a further experiment by Koo et al. [100], genipin was also shown to have an antiangiogenic effect, when assessed in the chick embryo chorioallantoic membrane assay. Moreover, data from lipopolysaccharide/interferon-γ (LPS/IFN-γ) in RAW 264.7 cells showing inhibition of NF-κB activation, nitric oxide (NO) production, and inducible nitric oxide synthase (iNOS) expression (50–300 μM) show the existence of an anti-inflammatory–antiangiogenic crosstalk. Hence, anticarcinogenesis, antiangiogenic effects, and direct effects on established cancer cells are all involved in the mechanism of action for these compounds. The overall gross mechanism of action of these compounds is depicted in Figure 3.

## 5. Lessons on the Mechanisms of Action

### 5.1. Mechanisms Related to Cell Cycle Regulation

Induction of apoptosis by anticancer agents is one of the common and rather gross mechanisms leading to cellular morphology and biochemical alterations and death. The induction of apoptosis and the inhibition of cellular proliferation by geniposide and genipin have been shown to be coupled with cell cycle arrest [38]. For example, G2/M phase arrest along with the induction of cyclin-dependent kinase inhibitor p21 (p21) and p21-dependent cyclins were shown to be induced by genipin in AGS human gastric cancer cells. As one expects, signalling pathways associated with this process are inevitably affected by genipin, and the transcription factor early growth response-1 (Egr1)-p21 crosstalk (both elevated by genipin at protein and mRNA levels) is among the mechanisms already reported. Egr1, as a transcription factor, could upregulate p21 by binding to the p21 promoter, following its translocation in the nucleus. Data by Ko et al. [38] thus gave some clue on the mechanism of apoptosis induction by genipin via caspase 3 and a p53-independent mechanism in the Egr1–p21 signaling pathway. They also showed that genipin increased p21 promoter activity and the interaction between Egr1 and the p21 promoter site in a dose-dependent manner. By inhibiting the activity of cyclin-dependent kinases, which are required for cell cycle progression, p21 induces cell cycle arrest at either the G1/S phase or the G2/M phase. The other most important mechanism observed in the study was the generation of ROS and the increase in mitochondrial permeability following genipin treatment. Mitochondrial permeability could predispose cells to necrotic and apoptotic cell death (see the following sections). Depending on the cell type and concentrations used, some differences in the cancer cell killing mechanisms may be evident. For example, Chang et al. [51] demonstrated an increase in *p53*, *c-Myc*, and B cell lymphoma gene 2 (*BCL2*) associated X (*Bax*) coupled with a decreased protein levels of B cell lymphoma gene 2 (Bcl-2) associated with the cytotoxic effect of penta-acetylated geniposide in C6 glioma cells. The cell cycle arrest reported was at G0/G1 at 0.3 mM, a dose slightly higher than the IC_50_ value (0.2 mM-52% cell viability inhibition).

A mechanism involving increased phosphorylation of p38 MAPK in genipin-induced apoptosis was demonstrated in non-small-cell lung cancer H1299 cells, via a mitochondrial apoptotic cascade [41]. Hence, increased levels of Bax and suppression of Bcl-2, coupled with the activation of the mitochondrial execution pathway through caspase-9 and -3 activations were reported after genipin treatment. The cell cycle arrest at the G2/M phase reported by these authors was also in line with what has already been discussed above, reported in other studies [38]. The downregulation of Bcl-2, the upregulation of Bax, and the proteolytic activation of caspase-3, along with the activation of JNK and p38 MAPK, were also established in MDA-MB-231 human breast cancer cells [47]. In human leukaemia K562 cells, the induction of apoptosis by genipin (200–500 μM) was coupled with upregulated Fas-L expression, increased caspase 3 activity, cell cycle arrest at the G2/M phase, and upregulated p-JNK, p-c-Jun, Fas-L, Bax, and cytochrome C [48].

Hwang et al. [43] studied the mechanism of action of genipin after hydrolysis of geniposide, as the latter appeared a very weak anticancer agent in vitro (see Section 4.1 above). In cancer cells such as DU145, MDA-MB-231, and U266, they showed that genipin could inhibit the constitutive signal-transducer-and-activator-of-transcription-3 (STAT3) activation by suppressing upstream Janus kinase 1 (JAK1) and c-Src. The expressions of Bcl-2, Bcl-xL, survivin, and cyclin D1 were downregulated, leading to cell cycle arrest at sub-G1 phase and apoptotic cell death. Cell cycle arrest at the G1 phase (along with increased levels of phosphorylated JNK, phospho-Jun, p53, and Bax proteins) was also observed in HeLa cells subjected to apoptosis by genipin [42]. In this regard, the effect of geniposide was consistent with that reported in various other studies, where anti-apoptotic gene products were suppressed. Given that STAT3, following activation, dimerizes and translocates into the nucleus to regulate the expression of various genes involved in cell survival, such as Bcl-2, Bcl-xL, and survivin, its inhibition could be a mechanism of apoptosis induction. For detailed insights into the subject, readers are directed to review articles [101,102] that outline constitutive STAT3 activation as a common biochemical marker of various cancer types and its targeting by potential chemotherapeutic agents. The cleavage of procaspase-8 and procaspase-9 could also be shown to be induced by genipin following inhibition of STAT3 activation, while caspase-3 was also activated [43]. JAK1 and c-Src are upstream protein tyrosine kinases that appeared to be inhibited by genipin along with STAT3 phosphorylation. Experiments by Lee et al. [49] also corroborated the above findings: In U266 and U937 cells, the suppressive effect of genipin on the constitutive STAT3 activation was shown to be mediated by the suppression of the activation of c-Src, but not JAK1. Furthermore, c-Src homology 2 domain-containing phosphatase-1 (SHP-1), which dephosphorylates and inactivates STAT3, was activated by genipin. In line with other studies (e.g., [43]), STAT3 target genes, such as Bcl-2, Bcl-x(L), survivin, cyclin D1, and VEGF, were downregulated.

Hong and Kim [44] examined the role of the mixed lineage kinase 3 (MLK3) in the ROS- and JNK-induced mitochondrial apoptosis in genipin-treated PC3 human prostate cancer cells. In these cells, sub-G1 cell cycle arrest, apoptotic cell death through activation of caspase, collapse of mitochondrial membrane potential, and release of cytochrome C were common features following genipin treatment. Genipin also stimulated MLK3 in these cells and generated ROS, processes that were both dependent on NADPH oxidase. As the phosphorylation of JNK and the induction of JNK by genipin were markedly inhibited in PC3-EGFP-MLK3 (K144R) cells expressing a dominant-negative MLK3 mutant, ROS- and MLK3-dependent apoptosis in these cells was suggested to be mediated through downstream activation of JNK. Another observation in this study relates to the fact that a specific inhibitor of p38 (PD169316) failed to suppress genipin-induced apoptotic cell death, hence highlighting the various possible pathways of apoptosis induction by this compound in different cell systems or at variable doses. On the other hand, penta-acetylated geniposide-induced cell death in C6 glioma cells was shown to be dependent on MAPK, including p38 (and also ERK and JNK) [52]. Sphingomyelinase (SMase)/nerve growth factor (NGF)/p75 activity in these cells, along with increased activity of the activator protein-1 (AP-1) and NF-κB and expression of FasL and caspase 3, have been shown to participate in cell death [52]. The NGF/p75 pathway appeared to be downstream of N-SMase/ceramide, while both were upstream of protein kinase Cδ (PKCδ) [53]. One must note that the experiments in this case were conducted at 0.6 mM penta-acetyl geniposide, a concentration far higher than that used in other studies, although lower concentrations (0.3 mM) have also been shown to induce the expression of PKCδ [55]. The study by Chang et al. [51] on C6 glioma cells apoptosis and cells cycle arrest at the G_0_/G_1_ (G_1_–S transition) phase by this compound (0.3 mM) similarly revealed p53 and c-Myc induction that also involved Bcl-2 family proteins, a decrease in the protein expression of cyclin D_1_, increased levels of cyclin-dependent kinase (cdk) inhibitor p21 protein, suppressed formation of cyclin D_1_/cdk 4 complex, inhibition of the phosphorylation of retinoblastoma (Rb), and dissociation of the Rb/E2F complex.

Overall, the induction of apoptosis by genipin and geniposide involved the activation of caspases that are known to mediate the common morphological changes, including DNA fragmentation, membrane blebbing, and the formation of apoptotic bodies, that were widely reported along with cell death. One common mechanism of action for these compounds appeared to be related to modulation of gene expression. Bcl-2 is a cell survival oncogene that prevents apoptosis, hence promoting cancer malignancies [103,104]. One of the various functions of the Bcl-2 family of proteins is the inhibition of cytochrome C release from mitochondria that triggers the apoptosis cascade. Hence, suppressing the expression of Bcl-2 predisposes cancer cells to increased apoptosis and cell death, as evidenced for various anticancer drugs. Other genes and proteins related to Bcl-2 are *BCL2* antagonist/Killer 1 (BAK) and Bax, which are pro-apoptotic and hence have the opposite function of Bcl-2, i.e., they induce the release of cytochrome C and other proteins and trigger apoptosis through caspase activation [105]. On the other hand, p53 is a tumor suppressor gene/protein that is also involved in the regulation of cell growth/death. As a transcription factor, p53 binds to DNA to control cellular activities, including the induction of DNA damage and cell cycle regulation leading to apoptosis. For this, p53 acts as transcription factor for genes coding for pro-apoptotic effector proteins and also orchestrate death signaling through the mitochondria and cytoplasmic cascades [106,107,108]. The transcription of enzymes involved in the repair of DNA is also induced by p53 [109]. In the various studies mentioned above for geniposide and analogues, modulation of the various cell survival/death regulatory genes/proteins has been demonstrated. The effect of genipin on signal transduction pathways, including those regulated by kinases, is similar to that of many clinically useful drugs. By inhibiting tyrosine kinases such as mitogen-activated protein kinase kinase (MEK1/2)-ERK1/2 signaling, the therapeutic potential of imatinib, gefitinib, and sunitinib has been validated [110].

The role of cell cycle regulation in the treatment of various cancers has been extensively reviewed (e.g., [111,112,113]). The cell division cycle is divided into distinct phases and include the Gap 0 (G_0_—resting stage), G_1_ (gap phase 1), synthesis (S) (DNA synthesis), G_2_ (gap phase 2), and M (mitosis) phases. Besides the role of various genes and protein kinases, the regulation of the cell cycle is under direct control of the various cyclins and cyclin-dependent kinases (cdks—which are serine/threonine kinases). D-type cyclins have been widely known for regulating cell cycle progression and tumorigenic cascades [114]. The interaction between cyclins and cdks is important in the smooth transition between the various stages of the cell cycle. Inhibitors, including those acting on p21 and various other associated proteins such as the nuclear transcriptional factors E2F-1 and Ets-1, also play important roles in cell cycle regulation. Various external stimuli that damage DNA, such as ionizing and UV radiations as well as many chemotherapeutic drugs, can induce inhibitors such as E2F1 [115]. The binding and/or activation of Cdk4 and Cdk6 with Cyclin D1 promotes G_1_/S-phase transition. Hence, the overexpression of cyclin D1 could promote tumorigenesis. The activation of p53 also leads to transcriptional downregulation of cell cycle proteins [116]. Another tumor suppressor protein in cancer cells is Rb which is inactivated through phosphorylation by the various cyclin cdks, leading to progression of the cell cycle through G_1_ into S. Hence, the effects of genipin and geniposide in the various studies discussed above as modulators of cell cycle arrest through pathways involving cyclins, cdks, and associated genes and signaling pathways outlined their possible mechanisms of action.

In view that the cytotoxic effects of genipin/geniposide occur at fairly large concentrations, multiple mechanisms may be involved. Gálvez et al. [117], for example, showed topoisomerase I poisoning by geniposide as another possible mechanism of action. Along with another iridoid glycoside, aucubin, the compound could stabilize the covalent attachment of topoisomerase I (but not topoisomerase II) subunits to DNA at sites of DNA strand breaks.

### 5.2. General Anti-Inflammatory Mechanisms

Carcinogenesis and inflammation have many overlapping signaling cascades in common. Key inflammatory mediators, such as (interleukin 1 (IL-1), IL-6, and TNF-α) are also implicated in carcinogenesis as well as in established cancer biology processes [118]. The expression of these cytokines is mediated through the activation of some common transcription factors, such as NF-κB, AP-1, STAT3, and Smad. Hence, while agents that chronically induce the expression of transcription factors such NF-κB and STAT3 could induce carcinogenesis [119,120], the same mechanism could be targeted by therapeutic agents to inhibit tumorigenesis. While chronic inflammation can increase the oncogenic potential of normal cells by its own, viral-induced carcinogenesis could also be mediated through enhanced oxidative stress and inflammatory mechanisms [121]. Besides cytokines, various chemokines and prostaglandins that are implicated in inflammation through their autocrine and paracrine effects are also known to participate in cancer biology [122]. Moreover, other carcinogenic agents such as UV irradiation do also trigger the inflammatory process in the body, including the induction of prostaglandins and cytokines that mediate carcinogenesis [123,124]. The link between cancer metastasis and inflammation is even clearer, as the process of angiogenesis and wound healing biology share many common features, and cancer metastasis also exploits the various biochemical and molecular sequels of leucocyte infiltration into extravascular tissues. For details of the crosstalk between cancer and inflammation, readers are directed to review articles on this subject [125,126,127,128].

On the basis of the above-mentioned cancer-inflammation crosstalk, the anti-inflammatory effects of genipin and geniposide are worth mentioning. Genipin has been shown to suppress the production of TNF-α both in vivo and in vitro [129]. In a cultured mouse macrophage-like (J774.1) cell line, 50 μg/mL (but not 10 μg/mL) could suppress TNF-α production, while mice pre-treated with genipin (100 mg/kg, i.p.) could be protected (53%) from the lethal dose of galactosamine/LPS. The antioxidant effect of genipin and geniposide that correlates with their antidiabetic effect has been well demonstrated (reviewed in [13]) and includes the induction of antioxidant HO-1 and GSH via induction of Nrf2 [130]. Through induction of HO-1, genipin can also inhibit TNF-α-induced vascular smooth muscle cell proliferation and migration [93]. The anti-inflammatory mechanisms of these compounds in the Alzheimer’s brain (e.g., [131,132] have been extensively reviewed recently [12]. The effect of genipin in gastrointestinal tract inflammation, including that induced by HCl-ethanol in an acute gastritis model, has also been established [40,133]. In the dextran sulphate sodium-induced colitis in mice, genipin was shown to display anti-inflammatory and protective effects against mucosal damage [134]. The suppressive effects on proinflammatory cytokines and NF-κB activation have were shown in both animal models and in vitro in LPS-activated Caco-2 cells [134].

The anti-inflammatory effects of genipin and geniposide have also been demonstrated through various other experimental models. By suppressing ERK1/2 signalling pathway, geniposide could suppress the inflammatory response in brain microvascular endothelial cells (Li et al., 2016) [135]. In LPF/IFN-γ stimulated murine macrophage (RAW 264.7) cells, genipin (50–300 μM) inhibited NO production and iNOS expression via NF-κB inhibition [136]. In vivo, both genipin and geniposide showed anti-inflammatory effects in carrageenan-induced rat paw oedema, carrageenan-induced air pouch formation, and affected NO content in the exudates [137]. These effects were demonstrated at doses from 50 to 400 mg/kg, with genipin being more potent than geniposide. Genipin (0.55–4.42 μmol/year) was also shown to have topical anti-inflammatory effects, as inhibition of the croton oil-induced ear oedema in mice was observed [136]. The antithrombotic activity of genipin and geniposide have also been widely reported (e.g., [138,139]). Both act through antioxidant and anti-inflammatory effects, exerting organoprotective effects in the neuronal and hepatic systems [140,141,142,143,144,145,146,147]. Hence, the known anti-inflammatory effects of these compounds could play part in abolishing the critical inflammatory component of cancer at various developmental stages (carcinogenesis, maintenance, and metastasis).

### 5.3. Cancer Cell Killing by Weaponizing Oxygen

Reactive oxygen species (ROS) include radical species such as superoxide anion radical (O_2_) and hydroxyl radical (OH·), and non-radicals such as H_2_O_2_. They are produced under normal physiological conditions through a variety of mechanisms, such the induction by cytokines, and under stress or inflammatory conditions. ROS are involved in the regulation of signal transduction in various cellular processes, including promotion of cell proliferation at submicromolar or micromolar concentrations. Higher levels (e.g., over 100 μM) of ROS are, however, known to induce apoptosis, while even greater concentrations, in the mM range, could induce rapid (necrotic) cell death within minutes because of cell membrane destruction. Numerous studies in the last few decades outlined that cancer cells development is dependent on low but chronic levels of ROS generation [148,149,150]. Hence, drugs that induce the generation of ROS intracellularly could have potential anticancer effects. In this connection, many existing anticancer drugs, such as the anthracyclines (e.g., doxorubicin, daunorubicin, epirubicin, and idarubicin), appear to work through such mechanisms [151]. Intriguingly, many compounds that are regarded as antioxidants, including a range of phytochemicals, are also known to induce the generation of ROS at low concentrations. Examples of antioxidant polyphenols inducing the generation of ROS include flavonoids [152,153], eugenol [154], quercetin [155], resveratrol [156], nordihydroguaiaretic [157], and curcumin [158]. We have also identified many other compounds that can induce pro-oxidative biological effects in the presence of copper ions, as well as cytotoxicity in cancer cells [159,160,161,162,163,164]. While the antioxidant effects of such compounds might explain their anticarcinogenic effects, their cytotoxicity and/or apoptosis induction is associated with ROS generation mechanisms. Even within the antioxidant polyphenols, however, some compounds and structural groups display more pro-oxidative effects than antioxidant effects and may also potentiate the cytotoxicity of other drugs (e.g., TNF-α) in cancer cells [163]. Interestingly, even known antioxidants such as vitamin E and ascorbic acids do also possess pro-oxidant effects [164,165,166] and mediate apoptosis through ROS mechanisms.

The generation of O_2_^−^ from molecular oxygen constitutes the initial step of ROS production in the mitochondria [167,168]. In immune cells, such as white blood cells, ROS generation primarily through NADPH oxidase is also a fundamental process in the body’s defence against pathogenic microorganisms and malignancy. The mitochondria are a source not only of ROS implicated in carcinogenesis and cancer cell killing by drugs but also of a range of proteins involved in cell cycle survival/death regulations. Hence, mitochondrial membrane damage and membrane potential are among the common parameters measured in apoptosis assays. A compromised mitochondrial membrane leads to leakage of proteins into the cytosol, including cytochrome C, to induce caspase activation. As outlined in the previous section, induction of apoptosis by genipin and geniposide through mitochondria-dependent or independent pathways has been shown to be associated with the generation of ROS in various cancer cell lines. One of the link between ROS and induction of apoptosis is the stress-activated protein kinase (SAPK) or JNK. When ROS are activated by drugs or any other stimuli (UV irradiation, heat shock, GSH depletion, and many chemotherapeutic drugs), the activation of JNK is initiated leading to induction of apoptosis. In the experiment by Kim et al. [45], genipin-induced apoptosis in hepatocarcinoma cells (Hep3B cells) was shown to be mediated by ROS/JNK activation of the mitochondrial pathway. JNK1/2 but not MEK1/2 nor p38 MAPK was shown to be activated. Transfection (of c-Jun) and inhibition studies (with NADPH oxidase) further confirmed the ROS generation-mediated apoptotic cell death via caspase-3 and JNK activation. These data were thus consistent with those from other studies highlighting the role of NADPH oxidase-dependent generation of ROS leading to downstream JNK activation as a mechanism of apoptosis induction by genipin. In light of comparative activity, genipin but not geniposide was able to induce the above events when tested at 200 μM concentration [45]. The role of JNK1/2, ERK1/2, and/or p38 MAPK following ROS generation could be dependent on the cell type and the type of stimulus. In this regard, the various signal transduction pathways associated with ROS generation induced by these drugs have already been described in the various cytotoxicity experiments listed above. The further role of ROS in the mitochondria is discussed in the following Section 5.4.

Cancer cell killing via the upregulation of ROS production is closely linked to depletion of antioxidant defenses. The diminished intracellular level of GSH is therefore the hallmark of cell death induced by ROS-activating agents. Thus, drugs that deplete GSH, such as plant isothiocyanates (e.g., moringin), do also have a potent anticancer effect [169]. Whether the α,β-unsaturated carbonyl moiety in genipin/geniposide discussed in Section 4.1 directly interacts with sensitive thiol groups to account for the diminished antioxidant (GSH) status remains to be proved.

### 5.4. Emerging Role of the Mitochondrial Uncoupling Protein-2 (UCP2) in Cancer Biology and Chemotherapy

**Figure 4:**
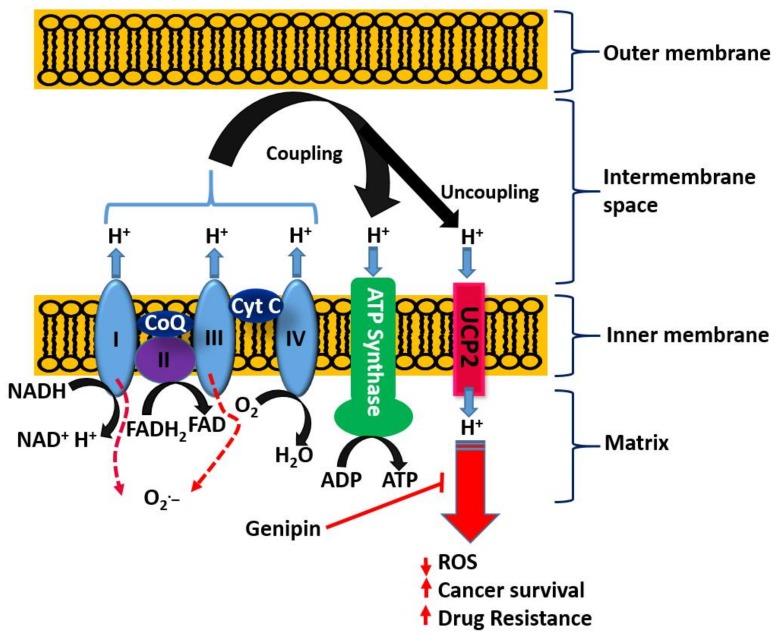
Targeting UCP2 by genipin in cancer. The electron transport chain in the mitochondria comprises complexes I–IV that transfer electrons from NADH through a series of oxidation–reduction reactions. The generation of the (H^+^) electrochemical gradient by the coordinated action of complexes (I, II, and IV) allows coupling with phosphorylation via ATP synthase. In addition to O_2_ serving as a final electron acceptor at complex IV, its premature reduction at complexes I and III could lead to O_2_^−^ formation. UCP2, which is excessively expressed in cancer cells, uncouples the process by creating a (H^+^) leak and reducing the mitochondrial membrane potential. This mechanism, exploited by cancer cells as a survival factor via reducing ROS generation, is targeted by genipin. CoQ, coenzyme Q and Cyt C, cytochrome C.

With respect to the role of the mitochondria in orchestrating both cancer development and death induced by a variety of agents, the uncoupling proteins (UCPs) have emerged as key players in recent years. The basic process of cellular respiration and/or oxidative phosphorylation in the mitochondria is based on the transport of protons (H^+^) out of the mitochondrial matrix to the intermembrane space. The resulting mitochondrial membrane potential and protons electrochemical gradient drive ATP synthase upon re-entry of protons. ATP generation in the mitochondria is therefore a result of the coupling of the electron transport chain to ADP phosphorylation to form ATP (Figure 4). Located at the inner mitochondrial membrane, the UCPs also transport protons back into the mitochondrial matrix and hence abolish the proton gradient required for ATP production, but they also diminish O_2_^−^ production (reviewed in [170]). As cancer cells are under increased oxidative stress, they require an increased activity of UCPs for their survival [171]. UCP2 is known to suppress mitochondrial ROS production and is employed by drug-resistant cancer cells to mitigate oxidative stress [172,173,174]. Hence, UCP2 is overexpressed in many cancer cells and plays a key role both in tumorigenesis and in cancer progression. One of the emerging rational approaches in targeting cancer cells by chemotherapeutic agents as well as in abolishing chemo-resistance is, thus, through UCP2 targeting. Once again, the overall therapeutic principle is based on the fact that a higher mitochondrial membrane potential means a higher level of ROS generation that could be overcome by the action of UPC2 as a natural antioxidant in cancer cells. Recent studies also suggest that overexpressed UCP2 in cancer cells remove Krebs-cycle metabolites from the mitochondria and hence shifts metabolic energy generation from mitochondrial Krebs cycle/oxidative phosphorylation to oxygen-dependent glycolysis: a fundamental process in cancer cells now widely known as the Warburg effect (Warburg, 1956) [175].

On the basis of the above discussion showing the critical role of UCP2 in cancer biology, the reported effect of genipin and analogues on this system appears to constitute a major anticancer mechanism of action. Mailloux et al. [176] studied the effect of genipin on UCP2 by using drug-sensitive HL-60 cells and the drug-resistant MX2 subline as model systems. First, they showed that a higher level of UCP2 could be detected in the mitochondria of drug-resistant cells, which appeared to account for 37% of the resting cellular oxygen consumption. The resting cellular respiratory rates were also higher in the drug-resistant cells. In the CHO cells stably expressing UCP2, genipin could suppress this respiration by ~22% as compared to no effects in empty-vector CHO cells not expressing UCP2. The increase in ROS by genipin was also shown to be linked to the inhibition of mitochondrial proton leak induced by UCP2. A study by Ayyasamy et al. [177] was consistent with the above findings, in that UCP2 was shown to be overexpressed in many cancer cell lines and to promote tumorigenic properties both in vitro and in vivo. In this environment, genipin was shown to downregulate both ROS and UCP2 function. The induction of apoptosis by genipin in pancreatic carcinoma cells (Panc-1) was also established to be mediated through UCP2 inhibition and subsequent ROS production [178]. A UCP2 inhibition-dependent mechanism of apoptosis induction in T47D breast cancer cells was reported by Cho et al. implicating the reduction of both glycolytic flux and mitochondrial oxidative respiration. [179]. A study by Yao et al. [179] was in support of the above finding, while a contradictory result was reported in a study by Ma et al. [180], where upregulation of UCP2 expression in HepG2 cell lines of hepatocyte steatosis was reported. This could be related to the differential effects of genipin at different doses, for example, in inducing or inhibiting the generation of ROS. Despite this discrepancy, genipin is now widely employed as a standard UCP2-inhibiting drug in various experimental models of ROS generation and in mitochondrial activity studies (e.g., [181,182,183,184,185,186,187,188,189] just to mention few). Many other studies on cancer based on UCP2 inhibition and ROS generation by genipin have also been published (e.g., [177,190,191]). The overall activity of genipin in this system is depicted in Figure 4.

The exact mechanism of action of genipin in inhibiting UPC2 is not known, but some clue on structure-activity relationships has been outlined by Yang et al. [178]. As geniposide (discussed in the previous section) is a weaker cytotoxic agent in vitro, and even 1-ethyl-genipin (ethyl insisted of glucose (geniposide) derivative) failed to show cytotoxicity, the authors suggested a crucial role of the free hydroxyl position both for cytotoxicity and UPC2 inhibition. Other derivatives, where the 10-hydroxyl position was derivatised (as acetate or trimethyl acyl) but still with the 1-OH hydroxyl group intact, were also shown to be active. Further research is however required to clearly elucidate the structure-activity relationship.

Readers should bear in mind that, while inhibiting UCP2 could offer a therapeutic option in cancer cells, it could also be detrimental in some pathological conditions. For example, downregulation of UCP2 by genipin was shown to exacerbate diabetes-induced kidney (proximal tubular cells) injury and apoptosis (Chen et al., 2014) [192]. Genipin could also exacerbate palmitate-induced hepatic steatosis through UCP2 inhibition [193]. Even though genipin is known to have a reputed antidiabetic effect (reviewed in [13]), its effect on UCP2 has been shown to be associated with reduced insulin-stimulated glucose uptake in 3T3-L1 adipocytes [194]. On the other hand, its effect on UCP2-mediated proton leak has been shown to reverse obesity- and high glucose-induced pancreatic beta cells [195].

## 6. Drug Potentiation

Through the effect related to UCP2 inhibition, Mailloux et al. [176] have shown that drug-resistant leukemic cells could be sensitized to the cytotoxic action of menadione, doxorubicin, and epirubicin when co-treated with genipin. Dando et al. [37] also studied the crosstalk between UCP2 inhibition and the ROS/Akt/mechanistic target of rapamycin (mTOR) axis for genipin/everolimus anticancer synergism. In their study, employing mice xenografts of pancreatic adenocarcinoma and in vitro experiments, inhibition of UCP2 by genipin triggered the Akt/mTOR pathway by a ROS-dependent mechanism. Tumour masses from mice injected with UCP2 (genipin) and mTOR inhibitors (everolimus) revealed a strong reduction in tumour volume and number of mitosis, associated with a marked cytosolic glycolytic enzyme glyceraldehyde 3-phosphate dehydrogenase (GAPDH) nuclear positivity. Their data appeared to reveal that genipin (200 μM) and everolimus could synergize in inhibiting cell proliferation both in vitro and in vivo through GAPDH nuclear translocation. The potentiation effect of genipin on anticancer agents in vitro via UCP2 inhibition and associated ROS generation has now been established for various drugs, including for the cytotoxic effect of cisplatin in colon cancer cells [36], breast cancer cells (MCF-7 and T47D) exposed to several chemotherapeutic agents [196], and various pancreatic adenocarcinoma cell lines (PaCa44, PaCa3, Panc1, CFPAC1, T3M4, and MiaPaCa2) exposed to gemcitabine [197].

Hauang et al. [198] have studied the potential potentiation effect of geniposide on doxorubicin cytotoxicity in vitro by using drug-resistant human osteosarcoma (MG63/doxorubicin) tumour cells. At a concentration that does not affect cancer cell growth, geniposide was shown to reverse doxorubicin resistance in a dose-dependent manner. In MG63/DOX cancer cell-derived xenografts in nude mice, geniposide also appeared to enhance the efficacy of doxorubicin. The effect of geniposide in this potentiation was shown to be associated with the downregulation of P-glycoprotein expression. A preliminary report by Su et al. [199] also showed an enhancement in the anticancer activity of rapamycin by genipin.

Genipin was also shown to potentiate the cytotoxic effect of chemotherapeutic agents, such as bortezomib, thalidomide, and paclitaxel in U266 cells [49]. On the other hand, by suppressing oxidative stress and inflammation, genipin can also attenuate cisplatin-induced nephrotoxicity [200]. In a murine model of cisplatin-induced nephropathy, genipin pre-treatment was shown to alleviate renal tissue injury by diminishing the serum blood urea nitrogen, creatinine, and cystatin C levels, as well as those of kidney injury molecule-1. Furthermore, genipin could attenuate cisplatin-induced oxidative/nitrative stress [200]. Hence, both the pro-oxidant and antioxidant effect of genipin and related compounds could be exploited for cancer and numerous other pathological conditions. Depending on concentrations/doses and cell types, variable outcomes could also be achieved.

## 7. General Summary and Conclusions

**Figure 5:**
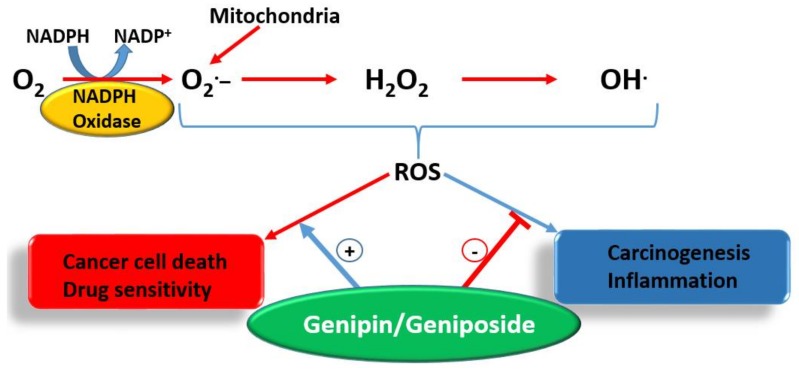
The dual effect of genipin/geniposide on ROS. The plus (+) sign indicates potentiation while minus (−) sign indicates inhibition.

Plants are widely exploited natural sources of drugs for cancer therapy. While some of these drugs have been identified through random screening programs, like taxanes, some resulted from research on traditional medicinal uses, as exemplified by genipin and geniposide presented in this review. The level of anticancer effect by genipin/geniposide in terms of potency does not match that of taxanes or other mechanism-specific anticancer drugs, but their multiple mechanisms of action and chemical characteristics appear to provide valuable lessons in advancing our knowledge in the field. These compounds have a plethora of effects in cancer development (carcinogenesis), survival, and metastasis that may be summarized as follow:

- Anticarcinogenic effect via antioxidant and anti-inflammatory (e.g., Nrf2, GPx induction) mechanisms.
- Targeting specific enzymes (e.g., GGT, MMPs) involved in carcinogenesis.
- Modulation of signal transduction pathways (e.g., MAPK such as JNK, p38, and ERK; PI3K, Akt, JAK1, etc.) involved in cell proliferation, inflammation, and cell death.
- Suppression of the production and function of proinflammatory cytokines (such as IL-1, IL-6, and TNF-α) and other proteins (iNOS).
- Modulation of various transcription factors (Egr1, NF-κB, AP-1, p21, STAT3) involved in inflammation and cancer biology and of transcriptional modulators such as SMAD2.
- Upregulation of genes/proteins that promote cell death and downregulation of survival genes/proteins; p53, Bcl-2, Bcl-xL, survivin, c-Myc, Bax, etc. are classical examples.
- Enhancement od ROS formation both by the NADPH oxidase and UCP2 pathways (see Figure 5).
- Triggering of cell cycle arrest (G1/S phase or G2/M phase) by modulating cyclin-dependent kinases.
- Mechanism related to topoisomerase I poisoning for cytotoxicity and downregulation of P-glycoprotein that allow drug potentiation and/or combination therapy.
- Activation of procaspases (e.g., procaspase-8 and 9) and caspases including the final apoptosis executioner, caspase 3.

The most crucial effect of genipin/geniposide appears to be linked to the double-edge sword mechanism of life and death balancing act by ROS and/or inflammation (Figure 5). They appear to enhance ROS generation both through the NADPH oxidase system and via the mitochondria, primarily through a UCP2 mechanism in cancer cells. This effect, particularly by genipin, supports thier use as gold-standard reference compounds in cancer pharmacology studies. The same mechanism involved in carcinogenesis is also targeted by genipin/geniposide, as evidenced from both in vitro and in vivo data. Such an effect, perhaps obtainable even at smaller doses, appears to have a relevant value for therapeutic approaches focused on the chemoprevention or nutraceutical utilization of plant resources. In this connection, the common fruits of the plants yielding genipin/geniposide are important resources to be taken into consideration. The demise of genipin/geniposide as anticancer agents appears to lie on their dual prooxidant/antioxidant effect, with their overall anticancer effect on established cancers appearing to be mediated at fairly large doses. Future studies are therefore required to disentangle these conflicting pharmacological properties, perhaps through structural design, to confer these compounds a far greater potency. In the meantime, the lessons learnt from these compounds as anticancer agents, from their pharmacokinetics profiles to their mechanisms of action, are further examples of the role played by plants as valuable sources of anticancer drugs.

## Abbreviations

AP-1	Activator protein-1
AFB_1_	Aflatoxin B_1_
Akt	Protein kinase B (PKB)
αSMA	α-Smooth muscle actin
ALT	Alanine aminotransferase
AST	Aspartate aminotransferase
BAK-1	*BCL2* Antagonist/Killer 1
Bax	*BCL2*-associated X
Bcl-2	B cell lymphoma gene 2
Cdk	Cyclin-dependent kinase
c-Fos	C-Jun, c-Myc, and c-Src; proto-oncogenes
EBV	Epstein-Barr virus
ECM	Extracellular matrix
Egr1	Early growth response-1
ERK1/2	Extracellular signal-regulated kinases 1 and 2
GAPDH	Glyceraldehyde 3-phosphate dehydrogenase
GGT	γ-Glutamyl transpeptidase
GPx	Glutathione peroxidase
GSH	Glutathione
GST	Glutathione-*S*-transferase
γ-GT	γ-Glutamyltranspeptidase
HO-1	Haem oxygenase-1
H_2_O_2_	Hydrogen peroxide
IFN-γ	Interferon-γ
IL-1	Interleukin 1
IL-6	Interleukin 6
iNOS	Inducible nitric oxide synthase
JAK1	Janus kinase-1
JNK	C-Jun-NH2-kinase
LPS	Lipopolysaccharide
MAPK	Mitogen-activated protein kinase
MEK1/2	Mitogen-activated protein kinase kinase 1 and 2
MLK3	Mixed lineage kinase-3
MMP-2	Matrix metalloproteinase-2
MT1-MMP	Membrane type I matrix metalloproteinase
mTOR	Mechanistic target of rapamycin
NF-κB	Nuclear factor kappa B
NGF	Nerve growth factor
NO	Nitric oxide
p21	Cyclin-dependent kinase inhibitor p21
PKCδ	Protein kinase Cδ
PI3K	Phosphoinositide 3-kinase
Rb	Retinoblastoma
ROS	Reactive oxygen species
SHP-1	c-Src homology 2 domain-containing phosphatase-1
SMAD2	Mothers against decapentaplegic homolog 2 also known as SMAD family member 2
SMase	Sphingomyelinase
STAT3	Signal-transducer-and-activator-of-transcription-3
TGFβ1	Transforming growth factor β1
TIMP-1	Tissue inhibitor of matrix metalloproteinase-1
TNF-α	Tumour necrosis factor-α TPA, 12-*O*-Tetradecanoylphorbol-13-acetate
VEGF	Vascular endothelial growth factor
